# Sequential, within‐season infection with influenza A (H3N2) in a usually healthy vaccinated child

**DOI:** 10.1111/irv.12668

**Published:** 2019-06-26

**Authors:** Jonathan L. Temte, Amra Uzicanin, Maureen Goss, Lily Comp, Emily Temte, Shari Barlow, Erik Reisdorf, Peter Shult, Mary Wedig, Kelsey Florek

**Affiliations:** ^1^ University of Wisconsin Madison Wisconsin USA; ^2^ Centers for Disease Control and Prevention Atlanta Georgia USA; ^3^ Wisconsin State Laboratory of Hygiene Madison Wisconsin USA

**Keywords:** case reports, coinfection, H3N2 subtype, influenza, viral surveillance

## Abstract

Cocirculation of varying influenza types, strains, and lineages allows coinfection and intra‐season sequential infection, although a same‐strain sequential infection has not been previously described. This case report describes the first known case of sequential laboratory‐confirmed influenza A (H3N2) infections in a child within one season.

## 1 INTRODUCTION

Cocirculation of distinct influenza types, subtypes, and strains has allowed for rare coincidental infection with more than one type or subtype of influenza.^1^, ^2^, ^3^ In addition, consecutive infection with influenza A and B viruses within a single season has been demonstrated.^4^ Recurrent within‐season influenza A (H1N1) infections have also been reported for two immunocompetent, but high‐risk patients.^5^ Here, we report a unique case of two sequential, within‐season infections with highly similar influenza A (H3N2) viruses in a usually healthy vaccinated child.

## 2 METHODS

This case was identified within a community‐based research study, occurring outside of medical care settings. Briefly, since January 5, 2015, a longitudinal school‐based influenza surveillance study has been ongoing at the Oregon School District (OSD) in South Central Wisconsin. ORCHARDS (ORegon CHild Absenteeism due to Respiratory Disease Study) is designed to assess the utility of school absenteeism monitoring for early influenza outbreak detection by comparing illness‐related absenteeism with medically attended influenza in surrounding communities. Parents of children with influenza‐like illness (ILI) symptoms voluntarily call a study hotline; students are screened for ILI criteria and, if eligible, receive a home visit from a research team member. Research staff collect nasal and naso/oropharyngeal (NP/OP) specimens along with symptom and epidemiologic data. Specimens are tested by rapid influenza diagnostic testing (RIDT; Quidel Sofia Influenza A + B FIA) within 2 hours of the home visit.

All specimens are then tested for influenza by rRT‐PCR (IVD CDC Human Influenza Virus RT‐PCR Diagnostic Panel) and other respiratory viruses (Luminex NxTAG Respiratory Pathogen Panel: RPP) at the Wisconsin State Laboratory of Hygiene (WSLH). The WSLH undergoes a rigorous validation process for diagnostic tests, including rRT‐PCR, in accordance with federal CLIA licensing standards. Unidirectional workflow, preparing aliquots of one specimen at a time, dedicated specimen processing equipment and laboratory area, and decontamination of biosafety cabinet (BSC) and laboratory benches after each use ensure cross‐contamination risks are minimized.

Beginning in September 2016, families of ORCHARDS subjects were invited to participate in a home transmission substudy, for which nasal swabs are obtained from family members the day of the visit (day 0) and 7 days later, along with a day 7 nasal swab from the ORCHARDS subject. All specimens from household members are tested for influenza using rRT‐PCR as above.

Exceptionally for this case study, next‐generation genome sequencing was performed at WSLH using the Illumina MiSeq™ platform. Sequence reads were trimmed using Trimmomatic with a 4‐bp sliding window quality score cutoff of Q30.^6^ For both samples, we mapped the trimmed sequence reads against a vaccine strain HA reference sequence A/Hong Kong/4801/2014 using Geneious version 11.0.5.^7^

The University of Wisconsin Institutional Review Board (IRB) (2013‐1357‐CP022) approved the study protocol, and the Centers for Disease Control and Prevention's Human Research Protection Office reviewed and approved deferral to the University of Wisconsin's IRB.

## 3 CASE PRESENTATION

### 3.1 Episode 1

**Figure 1:**
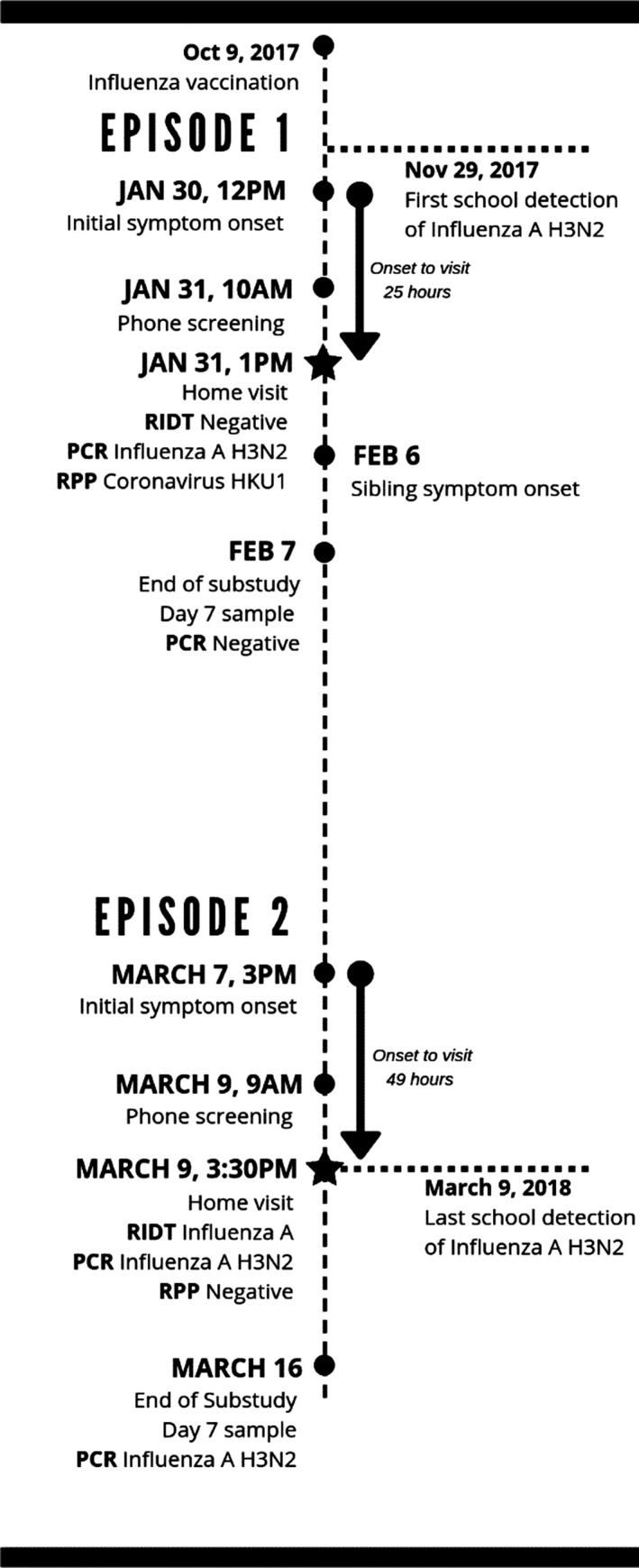
Timeline of events related to two sequential episodes of influenza A (H3N2) in a previously healthy, 4th‐grade child. (PCR, polymerase chain reaction; RIDT, rapid influenza diagnostic test; RPP, respiratory pathogen panel)

On January 31, 2018, a usually healthy, 9‐year old, non‐Hispanic white female was screened for ORCHARDS eligibility (Figure 1). She was a vaccinated (quadrivalent inactivated influenza vaccine on October 9, 2017) 4th‐grade student who lived with both parents and two younger siblings. All family members had received the 2017/2018 influenza vaccine. A home visit was conducted 25 hours after symptom onset. Her moderate ILI was characterized by fever (measured temperature = 37.2°C after antipyretic), chills, cough, sore throat, rhinorrhea, nasal congestion, headache, malaise, myalgia, anorexia, and sneezing. There was no recent travel, exposure to farm animals, or identified sick contact preceding onset. Nasal and oropharyngeal specimens were collected. Although the RIDT result was negative, rRT‐PCR was positive for influenza A (H3N2) (cycle threshold value [Ct] = 31.48) and RPP was positive for coronavirus HKU1. At follow‐up, she reported 2 days of absenteeism and continued cough, rhinorrhea, sore throat, myalgia, and headache. There had been no medical follow‐up or antiviral treatment. The rRT‐PCR result from the subject's day 7 swab was negative for influenza. No other family members reported illness on day 0, but one sibling developed mild respiratory illness on day 6. All family members tested negative for influenza by rRT‐PCR on day 0 and day 7.

### 3.2 Episode 2

On March 9, 2018, 37 days after the initial encounter, the subject was again screened due to a new ILI episode. A home visit was conducted 49 hours after symptom onset. Now 10 years old, the subject complained of significant symptoms including fever (measured temperature = 37.6°C after antipyretic), chills, cough, wheezing, nasal congestion, headache, malaise, myalgia, anorexia, burning eyes, and dizziness. Again, there was no recent travel, exposure to farm animals, or identified sick contact preceding onset. The subject's RIDT result was positive for influenza A, and rRT‐PCR results confirmed the presence of influenza A (H3N2) (Ct = 26.48). At follow‐up, she reported 2 days of absenteeism and continued fever, chills, cough, rhinorrhea, malaise, myalgia, headache, and anorexia. There had been no medical follow‐up or antiviral treatment. The rRT‐PCR results from the subject's day 7 swab continued to show influenza A (H3N2) (Ct = 33.30). No other family members reported illness on day 0 or day 7. All family members tested negative for influenza by rRT‐PCR on day 0 and day 7.

### 3.3 Whole‐genome sequencing

The subject's initial specimen from each episode, collected on January 31 and March 9, 2018, was prepared for full genomic sequencing at the WSLH. Both A (H3N2) strains were of the hemagglutinin gene clade 3C2.a2. After WSLH performed a consensus sequence comparison between the two viruses collected from the subject, three single nucleotide polymorphisms (SNPs) were identified within the coding region of the HA protein. Only one SNP was non‐synonymous and resulted in an isoleucine to leucine change at position 67 (in HA1). This particular polymorphism was near 100% frequency in the sequence reads and was four amino acids away from an established antibody epitope site (Site E).^8^ Both sequences (first isolate ORCH00001 and second isolate ORCH00002) were submitted to the GenBank^®^ genetic sequence database and given accession numbers (MK262897 and MK262898, respectively).

## 4 DISCUSSION

This is the first known case of two sequential, within‐season infections with influenza A (H3N2). Moreover, this case occurred within an immunocompetent and fully vaccinated child. The period between ILI episodes (37 days), the absence of influenza by PCR at 7‐day follow‐up of the first episode, the resolution of symptoms, and the development of a new ILI support two distinct episodes of influenza infection.

In the first episode, coinfection with coronavirus HKU1 was documented as well as a high Ct value for influenza A (H3N2) and a falsely negative RIDT. This may imply a relatively low viral load and possible immune interference induced by coinfection with coronavirus.^9^ Although the estimated vaccine effectiveness during the 2017‐2018 influenza season was low at 17% (95% CI: −14 to 40), the seasonal vaccine may have offered some partial protection.^10^, ^11^, ^12^

In the second episode, the subject's ILI was more significant, the RIDT was positive, and her initial specimen manifested a much lower Ct value, implying a higher viral load. This occurred despite vaccine receipt and prior infection with the same influenza subtype. The timing of the first episode, relative to the second, was such that neutralizing antibodies should have been present.^13^ Although the two viruses were genetically similar and clustered with other viruses that circulated within the community, it is possible that the I67L polymorphism could impart a structural change affecting antibody recognition. This case suggests that an influenza virus can evade the immune response of an immunocompetent, vaccinated child who was recently infected with a similar virus.

It is known that influenza remains under‐detected by established surveillance systems, which rely on sentinel‐based reporting of medically attended cases.^13^ This case demonstrates the intrinsic value of longitudinal studies of influenza within communities coupled with laboratory support that can provide antigenic characterization and genomic sequencing. A focus on medically attended influenza would have missed this case. Cocirculation of clade 3C2.a2 variants within a single community causing reinfection may offer a possible explanation for the prolonged and intense influenza season experienced in 2017‐2018. Further assessments are needed to assess the frequency of within‐season reinfection with influenza A (H3N2).

## CONFLICT OF INTEREST

The authors have no conflicts of interest to declare.
